# Workplace violence against healthcare workers in Pakistan; call for action, if not now, then when? A systematic review

**DOI:** 10.1080/16549716.2023.2273623

**Published:** 2023-11-08

**Authors:** Syeda Tayyaba Rehan, Mishal Shan, Syed Hasan Shuja, Zayeema Khan, Hassan Ul Hussain, Rohan Kumar Ochani, Asim Shaikh, Iqbal Ratnani, Abdulqadir J. Nashwan, Salim Surani

**Affiliations:** a Department of Medicine, Dow University of Health Sciences, Karachi, Pakistan; b Department of Pediatrics, Dr. Ruth KM Pfau Civil Hospital, Karachi, Pakistan; c Department of Medicine, The Aga Khan University, Karachi, Pakistan; d Department of Anesthesiology and Critical Care, Methodist DeBakey Heart & Vascular Center, Houston Methodist Hospital, Houston, TX, USA; e Department of Nursing, Hamad Medical Corporation, Doha, Qatar; f Adjunct Clinical Professor, Texas A&M University, Corpus Christi, TX, USA

**Keywords:** Healthcare, workplace violence, mental health, physical health, verbal abuse

## Abstract

**Background:**

Workplace violence (WPV) is a global problem that affects healthcare workers’ physical and mental health and impairs work performance. Pakistan’s healthcare system is not immune to WPV, which the World Health Organization recognises as an occupational hazard.

**Objectives:**

The primary objective of this systematic review is to determine the prevalence of physical, verbal, or other forms of WPV in healthcare workers in Pakistan. Secondary objectives include identifying the associated risk factors and perpetrators of WPV.

**Methods:**

A systematic review of six electronic databases was conducted through August 2022. Studies were included if they met the following criteria: 1) healthcare workers (HCWs), including physicians, nurses, and paramedic staff working in the private or public sector of Pakistan; 2) exposure to physical, verbal, or any type of violence. Data were extracted and analysed for the prevalence of WPV, types of violence, associated risk factors, and perpetrators of violence.

**Results:**

Twenty-four studies including 16,070 HCWs were included in this review. Verbal violence was the most common form of violence levied, with its highest prevalence (100%) reported in Islamabad and lowest verbal violence prevalence (25%) in Karachi. Verbal abuse was preponderant against female HCWs, while physical abuse was directed more towards males. The most common perpetrators were patient attendants, followed by the patients.

**Conclusion:**

Our review determines a 25–100% prevalence of WPV against HCWs in Pakistani medical setups. This occupational hazard needs the attention of relevant authorities in the country to put protective enforcement policies in place. Large-scale surveys should be conducted to better gauge the current plight of HCWs in the nation.

## Introduction

Violence against healthcare workers (HWCs) is a frequently encountered quandary in hospitals. It takes many forms ranging from life-threatening targeted violence involving lethal weapons to long-lasting verbal abuse [1]. Workplace violence (WPV) affects HCWs’ physical and mental health, impairs work performance, and may culminate in undue medical errors [2–4]. According to Havaei et al., nurses who encountered violence had a 2–4 times higher probability of developing post-traumatic stress disorder (PTSD), anxiety, depression, and burnout as compared to nurses who had never experienced workplace violence [4]. Following a violent episode, more than half of the victims reported experiencing loss of self-esteem, humiliation, and other negative emotions in addition to anxiety and despair [5]. Acts of property and equipment damage impact patient care on an even larger scale [5]. Despite attempts to abate such incidences, they remain a significant cause of concern in our healthcare setup, being a barrier to optimal patient management. The World Health Organization (WHO) recognises it as an occupational hazard, and an alarming 62% of healthcare professionals are reportedly impacted by WPV [6].

**Figure 1.**
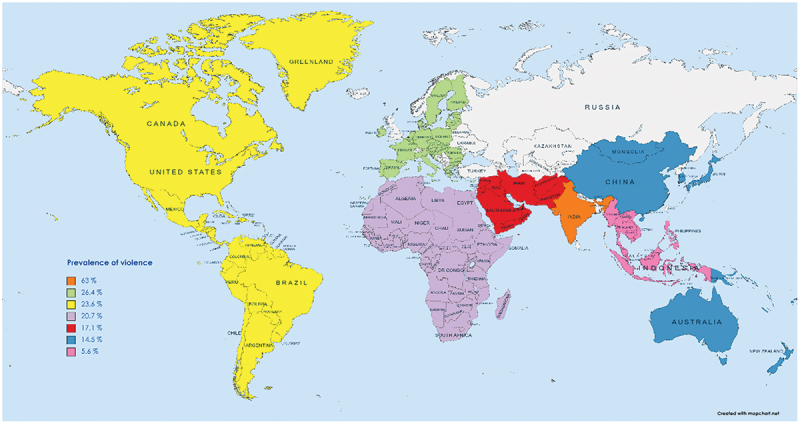
Prevalence of violence against healthcare workers in different regions [12].

The statistics for a developing nation like Pakistan are even more concerning since there is a continual battle to balance population demands with limited resources with a concurrent lack of security in hospitals, sub-standard law, and order conditions, and overcrowding of hospitals [7]. A report issued by the Pakistan Medical Association claimed that 128 doctors in Pakistan had lost their lives to violence from 1995–2015 [8]. A review of 253 studies by Vento et al. reported 61.9% of the participants to have suffered from some form of violence, with most incidents narrated from Asian and North American countries [9]. In Iran, 73% and 36% of emergency medical personnel reported being verbally and physically abused, respectively [10]. Similarly, in India, the frequency of violence against healthcare workers was estimated to be around 63% [11,12]. The prevalence of violence against HCWs in different regions is presented in Figure 1.

Numerous studies have been conducted nationwide to gauge the magnitude of the problem; however, they are largely limited by their single-centred study design. Therefore, in this comprehensive systematic review, we aim to quantify the prevalence of workplace violence and its associated risk factors in Pakistan by conducting a thorough systematic review.

## Methods

This systematic review has been conducted in compliance with the guidelines provided by Preferred Items for Systematic Review and Meta-Analysis (PRISMA). The review has been registered on PROSPERO [13]. [Registration ID: CRD42022344970]

### Information sources and search strategy

**Figure 2.**
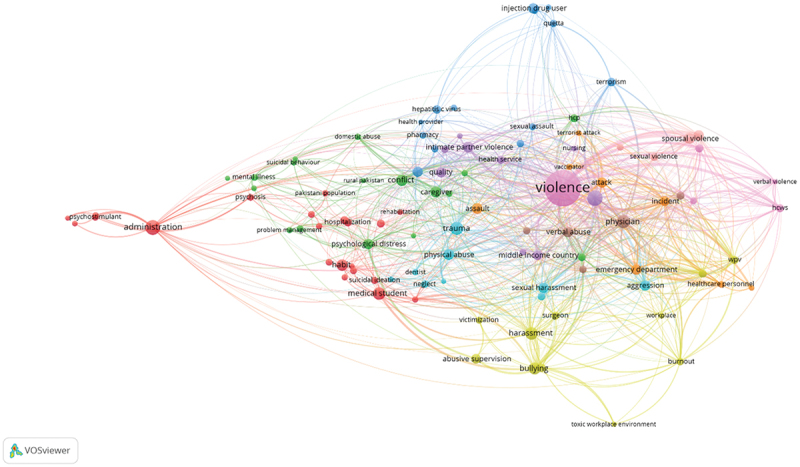
PubMed keywords network map based on relevance [14].

We queried electronic databases (MEDLINE, OvidEmbase, Cochrane CENTRAL, Science Direct, ERIC, and Google Scholar) from their inception till August 2022 by using medical subject headings (MeSH) ‘healthcare workers violence’ OR ‘workplace violence’ OR ‘healthcare abuse’ OR ‘healthcare worker’ AND ‘Pakistan’ OR ’Pakistani hospitals’ OR ‘Pakistani clinics’ without any time or sample size restrictions. The search string has been amended and reoriented accordingly for each search engine. Pakmedinet was also used to further search to incorporate grey literature. The complete search strategy used in each of the databases is given in Supplementary Table S1. The PubMed keyword relevance map is presented in Figure 2.

### Inclusion criteria

We used the following inclusion criteria to formulate research questions for this review:

- **Population**: HCWs, i.e. doctors, nurses, technicians, support staff, administration, ambulance staff, vaccinators, lady health workers (LHWs), midwives, nursing aids, porters, guards, unit receptionists, and housekeepers.
- **Intervention**: Individuals exposed to violence which include
  1. Physical abuse, sexual abuse, and verbal abuse. Threat to assault, physical assault.
  2. Intimidation, aggression, weaponry attacks.
  3. Facility or property damage, robbery, extortion, and theft.
- **Comparator**: None
- **Outcomes**: The primary outcome of interest was the prevalence of physical, verbal, or any other form of violence, including sexual violence and facility damage against healthcare personnel. Secondary outcomes included the role of job category, gender, age, working experience, type of hospital (i.e. private, or public), and department of work on violence faced by HCWs.
- **Study design**: Observational studies (cross-sectional and quasi-experimental studies)

### Exclusion criteria

Studies conducted in countries other than Pakistan, duplicate records, and articles in languages other than English were excluded. Case reports, commentaries, and editorials were also excluded.

### Study selection

Retrieved articles from the systematic search were imported into EndNote Reference Manager (Version X7.5; Clarivate Analytics, Philadelphia, Pennsylvania), where duplicates were removed. All articles were carefully screened by two independent reviewers (MS and SHS). Non-relevant studies were eliminated based on title and abstract. Full texts of articles were then comprehensively reviewed for inclusion based on predetermined criteria. A third reviewer (STR) was consulted to resolve discrepancies.

### Data extraction

Two reviewers (MS, ZK) independently abstracted data from the selected studies by using a self-designed Microsoft Excel sheet, and discrepancies were resolved through feedback from a third reviewer (STR). Data on study year, region, design of the study, health facility type, sample size, age, gender, type, mode of assessment, and prevalence of violence was extracted from all included articles.

### Quality assessment

The quality assessment for cross-sectional studies was conducted using the Newcastle Ottawa Quality Assessment Scale [15], while the Joanna Briggs Institute (JBI) tool for appraisal of quasi-experimental studies [16] was used to perform the quality assessment of quasi-experimental studies. Two researchers (MS and STR) individually carried out the quality assessment, and any discrepancies were resolved after discussion. The Newcastle Ottawa Quality Assessment Scale assigns grades to studies based on three factors (selection, comparability of study groups, and the outcome of interest). A study can receive a possible highest rating of 9 for cross-sectional studies. Cross-sectional studies with a total score of 8 or 9 points were deemed to have a low risk of bias; studies with a score of 6 or 7 points were judged to have a moderate risk of bias, and studies with a score of 5 points or less were regarded to have a high risk of bias. The Joanna Briggs Institute (JBI) tool for appraisal of quasi-experimental studies assigns a score of 1–9 to articles based on a 9-point questionnaire. The studies were thus categorised as low (points 1–4), moderate (5–7), and high (8–9) quality.

### Data synthesis

The outcomes of included studies were qualitatively synthesised and not pooled for meta-analysis due to the different methodological approaches used in assessing them. Study findings are summarised in the results section and tabulated in summary tables.

## Results

### Literature review

**Figure 3.**
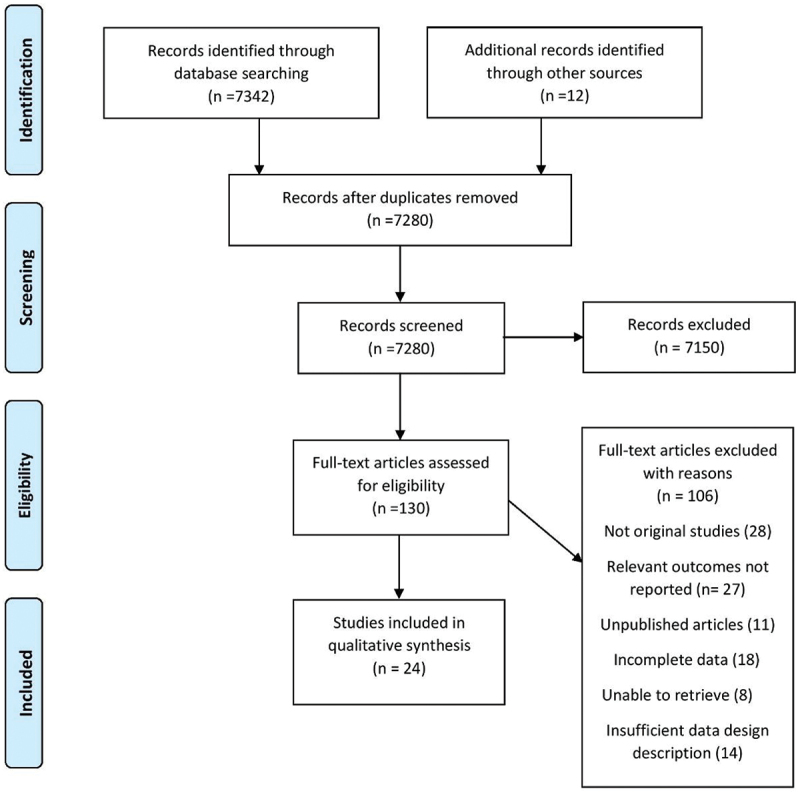
Summary of literature search.

Our initial literature search on six different electronic databases yielded 7342 prospective articles. After removing duplicate articles and abstract screening, 130 articles were selected for full-text review. Further, 106 articles were removed after a full-text review for not meeting our inclusion criteria. Finally, 19 cross-sectional [17–35], 2 Quasi-experimental [36,37], 2 mixed-methods studies [38,39], and 1 qualitative investigation study [40] were included. A summary of the literature search is presented in the PRISMA flow chart (Figure 3). Study characteristics and baseline characteristics of participants are provided in Supplementary Table S2 and S3, respectively.

### Study characteristics

A total of 24 studies, conducted from January 2010 till August 2022, including 16,070 HCWs, were included. Most of the individuals included in our studies were females. Seven (29%) studies evaluated the frequency of violence against doctors [25,27,28,31–33,35], seven (29%) studies against nurses [19,22,23,26,29,34,40], and ten (42%) studies against all types of HCWs, including guards, support staff, administration, and paramedics [17,18,20,21,24,30,36–39]. Eleven studies (46%) were conducted in Karachi [20,23,27,28,28–33,38], 3 (13%) in Peshawar [17,26,39], four (17%) in Lahore [19,21,24,34], two (8%) in Islamabad [22], one (4%) in Faisalabad [25], one (4%) nationwide [35], and two (8%) of the studies were conducted in more than one city [18,36]. Seventeen (71%) studies assessed the prevalence of both verbal and physical abuse. Imran et al. assessed the prevalence of verbal abuse, while Qadeer et al. assessed the prevalence of physical violence [21,32]. Seven (29%) of the included studies also assessed the frequency of sexual harassment faced by the HCWs [19,22,23,29,31,32,34]. Ten (42%) studies analysed the role of gender with the frequency of violence [18,20,23,27–29,31,32,35,38]. Five (21%) studies reported the association of violence with the age of HCWs [20,23,25,29,40]. Two (8%) studies assessed the association of violence with the type of hospital (i.e. private or public) [17,18]. Six (25%) studies evaluated the prevalence of violence in relation to working experience [20,23,28–30,32].

### Quality assessment

A total of 20 (83%) cross-sectional studies [17–35,40] and four (17%) quasi-experimental studies [36–39] were examined for bias. Of the 20 cross-sectional studies, four (20%) studies had a high risk of bias [21,26,34,40], six (30%) studies had a moderate risk of bias [22–24,31,32,35], and ten (50%) studies had a low risk of bias [17–20,25,27–30,33]. All four of the quasi-experimental studies were of high quality based on the JBI appraisal tool [36–39]. A summary of quality assessment is presented in Supplementary Tables S4 and S5.

### Results summary

**Figure 4.**
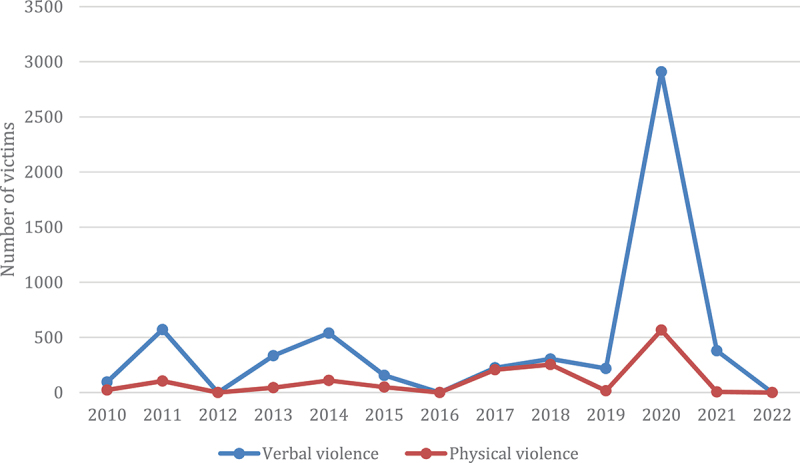
Annual growth rates of physical and verbal violence against HCWs in Pakistan [17,,23–27,29,,38,39].

**Figure 5.**
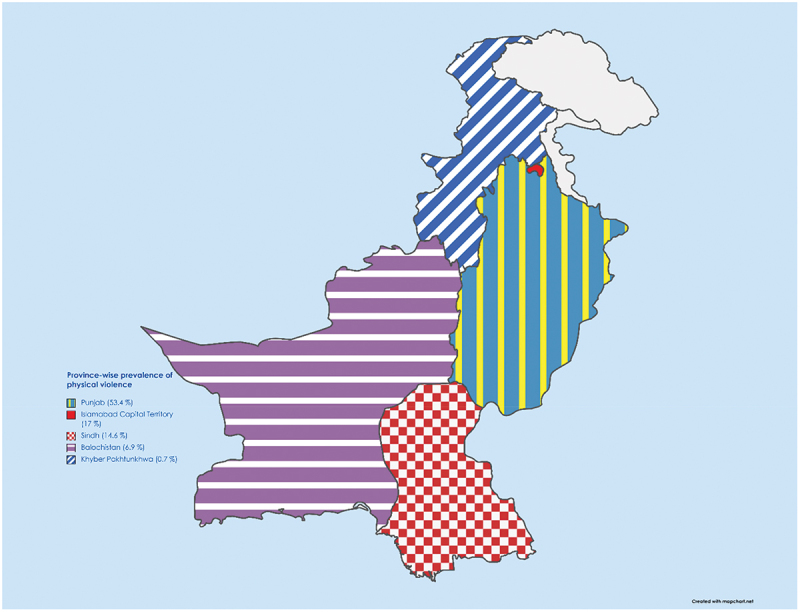
Provincial prevalence of physical violence against HCWs in Pakistan [17,19,22,38,41].

The highest prevalence of verbal violence (VV) was reported to be 100% by Shahzad et al. [40] in Islamabad, while the lowest VV prevalence was reported to be 25% by Siddiqui et al. [30] in Karachi. Regarding the highest prevalence of physical violence (PV), Qadeer et al. [21] reported a staggering 67% in Lahore, while the lowest prevalence of PV was 0.7%, reported by Khan et al. [17] in Peshawar. The annual growth rate of violence against HCWs is presented in Figure 4. The provincial prevalence of PV and VV is presented in Figures 5 and S1 respectively.

PV and VV Both followed a similar trend throughout the last 12 years, with victims of VV always dominating those of PV. The rise and fall in the number of victims of VV were more rapid as compared to PV victims. From 2010–11, VV increased sharply, while a gradual rise was evident in PV, followed by a decrease in both types of violence in 2012. In the following two years, the number of VV victims reached 539, while 109 victims faced PV, after which there was a decline in occurrences in 2015 and 2016. A similar trend of a 2-year increase followed by a 1-year decline was noticed till 2019, after which there was a huge inclination in 2020, followed by a fall in number in the last two years.

Eighteen (75%) of the included studies reported that the prevalence of VV was higher compared to PV. The majority of studies revealed that VV was preponderant against female HCWs [18,19,22,23,40] while PV was directed more towards male HCWs [35]. A study by Maheshwari et al. reported that the likelihood of WPV among male HCWs was higher compared to their female counterparts. However, this difference was not statistically significant [28]. The support staff was the most vulnerable group exposed to violence, followed by paramedics, nurses, and physicians [18]. Of the nurses who were exposed to violence, most of them were aged twenty to thirty, interns, or had an experience of <5 years [29,40]. However, according to Maheshwari et al.’s and Maaari et al.’s studies, HCWs (physicians, surgeons, chief medical officers, medical officers, PG residents, and house officers) and nurses who had >5 years of working experience reported greater exposure to violence [23,28]. A study conducted by Jafree et al. in Lahore also concluded that SV was more probable to occur among single Muslim nurses and those belonging to non-Punjabi ethnicity [19]. Workers in public healthcare facilities were more exposed to violence as compared to the private healthcare sector [17,18]. Departments of emergency were more prone to violent incidents as compared to the wards and outdoors [17,24,32]. Another finding outlined that male HCWs experienced more politically targeted violence in contrast to women [32]. VV was most frequent in morning shifts, and PV was during night shifts [19,40]. A significant association between VV and bullying was reported with the qualification of the HCWs [22]. The perpetrators were most commonly either the patient’s attendants or, less commonly, the patients themselves.

## Discussion

The prevalence of WPV among HCWs in Pakistan was examined in this systematic review. PV, VV, bullying, harassment, threats of assault, extortion, and facility damage were all described as different types of workplace violence by the studies reviewed. Congruent to our study, the greater frequency of WPV against HCWs in Asia compared to other regions was highlighted by Liu et al. in their meta-analysis of 253 studies from around the world [6]. The frequency of WPV in Africa was also examined by Njaka et al. in a systematic review, which revealed that verbal violence was by far the most common type of violence [42].

The frequency of WPV against HCWs in Pakistan has not been thoroughly and systematically studied, establishing this as the first review of its kind. In order to comprehend the genuine plight of WPV in hospitals in Pakistan, this review, which included 24 papers, has brought forth additional knowledge. We observed that the trend of verbal abuse was most prominent in all included studies. Our findings that VV and verbal threats are the most common kind of violence experienced in hospitals as supported by another scoping review by Hahn et al. [43]. Miscommunication between healthcare staff, particularly nurses and patients, is regarded as one of the main causes of the incidence of verbal abuse [44]. HCWs have also been habituated to verbal abuse, which is regarded as a ‘daily aspect of their employment,’ which explains the endemic prevalence of VV and why it continues to escalate [20]. The reporting rate of verbal aggression is extremely low since hospitals have not taken encouraging action against perpetrators in such cases [45]. According to survey respondents, there is a serious paucity of ‘no-tolerance policies’ at Pakistan’s tertiary care facilities, which perpetuates such violent episodes [38].

Our comprehensive review revealed a notable pattern of rising VV and PV prevalence between 2019 and 2022. The occurrence of the COVID-19 pandemic during this time period may have contributed to this finding. Bhatti et al. have emphasised the dramatic increase in WPV incidence in Pakistan during the epidemic [46]. Attendants’ frustrations with a lack of resources to treat and admit patients [47], death of patients [48], and refusal to hand over the patient’s dead body [46], protests by HCWs due to a lack of PPEs [49], and mob-related violence against HCWs during COVID-19 have all been cited as reasons for this rise [48]. Our review explored a unique finding which revealed a significantly lower incidence of WPV reported in Karachi despite it being the largest metropolis in the country housing three large tertiary care hospitals. This finding can be owed to under-reporting of WPV incidents in Karachi due to a lack of large government-led surveys to better understand the plight of HCWs in Sindh, especially in Karachi.

Among the papers included in the current investigation, gender differences were observed in multiple studies; compared to their male counterparts, female HCWs, such as nurses and doctors from the wards, ICU, and emergency department, were more likely to experience verbal abuse, physical assault, and sexual harassment [6,18,23,29]. Due to their propensity for a high level of WPV in hospitals, nurses have been the subject of it across a significant proportion of the literature. Liu et al.’s meta-analysis and comprehensive review support these conclusions, according to which nurses were the group of healthcare workers most likely to be exposed to WPV (22.99%; *p* = 0.0364) [6]. Female nurses being assigned night shifts, which has been demonstrated to be the most vulnerable time period for experiencing WPV, as well as under-resourced personnel, a high workload, and nurses’ lack of WPV awareness among healthcare professionals are antecedent factors that are connected with this common result [50–52]. Nurses in Pakistan are considered a marginalised section of society due to a lack of respect for the nursing profession [53]. The nature of the labour that nurses do, such as washing patients and changing their clothes, is severely looked down upon owing to cultural norms [29]. Because of a negative public impression, the nursing profession is seen as a lower socioeconomic career in Pakistan [54]. Additionally, the already existing class division in Pakistan encourages negative attitudes towards the nursing profession [55]. These factors, thus, significantly elevate the levels of WPV experienced by nurses.

WPV has been connected to a number of sociodemographic variables, including workplace, age, educational level, job history, and shift work, among others. According to Khan et al. [17] and Imran et al. [24], the emergency department is a common location for WPV, and emergency department physicians are also more likely to experience VV and PV [18,31,33,38]. This discovery is in line with the investigation conducted in Africa. This may be due to the emergent nature of the situation, the frequently chaotic nature of emergency facilities, and the subsequent emotional lability of patient family members [42].

There is a wide range of literature on the relationship between the training and work experience of doctors and nurses and violence in healthcare facilities [23,29]. More likely to become victims of violence of any kind have also been identified to be practitioners with employment histories longer than five years [23,28]. Contrastingly, our review showed that sexual harassment was more common among nurses with fewer than five years of experience [29]. Although Kahsay et al. studied working circumstances for nurses in which they are exposed to physical and sexual harassment [56], this link has not been explored in other related systematic and comprehensive investigations. This can be explained by the fact that nurses are subjected to physical and sexual harassment because they are more accessible to patients, caregivers, and visitors [56,57]. Additionally, women are already considered the vulnerable sex and hence fall victim to gender-based violence in South Asian settings, where nurses make up the large bulk of the nursing workforce [57]. In culturally patriarchal nations such as Pakistan, men are often more likely to be the primary perpetrators of violence [57], and there is a presence of oppressive attitudes towards women as well as dominant male hierarchies in workplaces. These cultural factors perhaps contribute to the additional burden of WPV experienced by women [19].

Lack of effective communication, unrealistic expectations, the perception of subpar care, and governance failures are the most often reported reasons for workplace violence against HCWs [17,18] Patient death, responses to critically sick patients, delays in care, and a lack of resources such as equipment or medications are other factors that contribute to WPV against HCWs [17,18]. The percentage of HCWs who chose to report violent episodes remains incredibly low, and even when reporting did occur, hospital administrations have been unable to provide adequate support [27,40]. Combating WPV against HCWs will require rigorous education programmes to provide widespread awareness regarding healthcare as a profession. Media can play a vital role in humanising the healthcare profession by running programmes which document the daily routine of the profession to better help the masses in understanding the struggles of this public health profession [45]. Healthcare regulators and government programmes can also contribute by establishing proper palliative care programmes to educate not only the families of the patients but also the doctors and nurses dealing with patients with long-term illnesses [45]. This can aid in bridging the communication gap between the HCWs and the attendants which is noted to be one of the biggest contributors of VPW.

### Implications for further research

In summary, the present research has solidified the prevalence of violence against healthcare workers in Pakistan. Each region’s sociocultural elements and violence are closely related. Most of Pakistan’s people live below the poverty line, forcing them to seek treatment at tertiary care facilities in big cities like Karachi, Lahore, Islamabad, and Peshawar. Due to limited funding, overworked, and underpaid staff, many healthcare facilities are under tremendous strain.

Due to the hostile atmosphere that is therefore nurtured, WPV occurrence increases in healthcare facilities. Doctors often fail to provide patients with enough one-on-one care in a timely manner, owing to resource deficiencies, which aggravates already strained physician-patient relationships [45]. WPV is an urgent threat to the occupational safety of HCWs, and local government organisations should have a zero-tolerance approach towards violence. To better understand the causes of WPV and develop strategies to combat them, extensive national surveys should be carried out. As communication barriers are known to have a significant role in the spread of violence [44], healthcare professionals should receive training and instructions on how to communicate better.

In order for nurses, workers, and physicians to be able to report such occurrences of violence to the relevant authorities with the management’s assistance, management failure at public hospitals should also be given additional consideration [44,45]. Given that nurses are most likely to be the targets of physical and sexual harassment, these findings highlight the urgent need to adopt protective rules and regulations for violence against nurses.

To prevent violent situations in the emergency room’s high-stress atmosphere, prevention methods are also required [58]. Coping techniques for WPV have been introduced in developed countries such as the United States and Canada [37,59]. These initiatives include the formation of an HCW union to better protect HCW rights [59]. In addition, a law was passed in Maryland that requires the formation of a workplace protection committee to prohibit verbal aggression and hostile behaviour towards HCWs [60]. There has also been a focus on equipping HCWs to protect themselves during such confrontations, including self-defence training [61]. In developed countries, these measures have been partially successful. Pakistani lawmakers should enact comparable policies and laws to ensure a safe working environment for HCWs.

### Strengths & limitations

This is the first comprehensive study of its kind that examines WPV throughout Pakistan. This study can prove to be crucial in determining the severity of WPV in Pakistani healthcare facilities because the included studies were carried out in various regions of the nation, including Sindh, Punjab, and Khyber Pakhtunkhwa. This systematic review’s thorough analysis of the interrelated social and cultural elements that contribute significantly to the persistence of workplace violence in the healthcare industry is one of its key strengths. This is consistent with international systematic reviews that have been published in places like Singapore [45].

This systematic review is not without its limitations. The vast majority of studies that were included were observational in style. The investigations’ use of a variety of instruments and questionnaires to gauge the prevalence of violence in the workplace could have produced inconsistent findings. Included studies’ observation periods were not uniform, which could have caused recollection bias. The primary weakness of the study was the heavy reliance on self-reporting techniques.

## Conclusion

In this systematic review, we have established a 25–100% prevalence of WPV against HCWs in Pakistani medical setup. We found that WPV is routinely carried out against physicians, nurses, and staff in wards, emergency departments, and ICUs. This occupational hazard needs the attention of relevant authorities in the country to put protective enforcement policies in place. Large-scale surveys should be conducted to better gauge the current plight of HCWs in the nation.

## Supplementary Material

Supplemental Material

## Paper context

*What was already known?*

Workplace violence is a global problem affecting healthcare workers’ physical and mental health and impairing work performance. Its prevalence and risk factors in Pakistan’s healthcare system are not well established.

*What’s New?*

This review analysed the prevalence and risk factors of workplace violence in Pakistan’s healthcare system, finding verbal violence to be the most common, with patients and their attendants as the most common perpetrators. The study emphasises the need for protective policies and interventions.

What are the *implications of the paper?*

This review emphasises the need for protective policies and interventions to address workplace violence in Pakistan’s healthcare system, including communication skills training and security measures to ensure healthcare workers’ safety.

## References

[1] Workplace violence in the health sector - country case study research instruments - survey questionnaire [Internet]. World Health Organization; [cited 2023 Jul 26]. Available from: https://www.who.int/publications/m/item/workplace-violence-in-the-health-sector—country-case-study-research-instruments—survey-questionnaire

[2] Alnofaiey YH, Alnfeeiye FM, Alotaibi OM, Aloufi AA, Althobaiti SF, Aljuaid AG. Workplace violence toward emergency medicine physicians in the hospitals of Taif city, Saudi Arabia: a cross-sectional survey. BMC Emerg Med. 2022 Apr;22:59. doi: 10.1186/s12873-022-00620-w 35392829 PMC8991560

[3] Workplace Violence in Healthcare [Internet]. Occupational safety and health administration; [cited 2023 Jul 26]. Available from: https://www.osha.gov/sites/default/files/OSHA3826.pdf

[4] Havaei F. Does the type of exposure to workplace violence matter to nurses’ mental Health? Healthcare. 2021 Jan 5;9:41. doi: 10.3390/healthcare9010041 33466294 PMC7824770

[5] Kaur A, Ahamed F, Sengupta P, Majhi J, Ghosh T, Joe W. Pattern of workplace violence against doctors practising modern medicine and the subsequent impact on patient care, in India. PLoS One. 2020 Sep 18;15:e0239193. doi: 10.1371/journal.pone.0239193 32946495 PMC7500628

[6] Liu J, Gan Y, Jiang H, Li L, Dwyer R, Lu K, et al. Prevalence of workplace violence against healthcare workers: a systematic review and meta-analysis. Occup Environ Med. 2019 Dec;76:927–11. 31611310 10.1136/oemed-2019-105849

[7] Khalid F, Abbasi AN. Challenges faced by Pakistani healthcare system: Clinician’s perspective. J Coll Physicians Surg Pak. 2018 Dec;28:899–901. 30501822 10.29271/jcpsp.2018.12.899

[8] Baig LA, Shaikh S, Polkowski M, Ali SK, Jamali S, Mazharullah L, et al. Violence against Health care providers: a mixed-methods study from Karachi, Pakistan. J Emerg Med. 2018 Apr;54:558–566.e2. 29449119 10.1016/j.jemermed.2017.12.047

[9] Vento S, Cainelli F, Vallone A. Violence against healthcare workers: a worldwide phenomenon with serious consequences. Front Public Health. 2020 Sep 18;8:570459. doi: 10.3389/fpubh.2020.570459 33072706 PMC7531183

[10] Sahebi A, Jahangiri K, Sohrabizadeh S, Golitaleb M. Prevalence of workplace violence types against personnel of emergency medical services in Iran: a systematic review and meta-analysis. Iran J Psychiatry. 2019 Oct;14:325–334. doi: 10.18502/ijps.v14i4.1984 32071607 PMC7007507

[11] Hossain MM, Sharma R, Tasnim S, Al Kibria GM, Sultana A, Saxena T. Prevalence, characteristics, and associated factors of workplace violence against healthcare professionals in India: a systematic review and meta-analysis. MedRxiv. 2020 Jan;3:2020–2021.

[12] Li YL, Li RQ, Qiu D, Xiao SY. Prevalence of workplace physical violence against Health care professionals by patients and visitors: a systematic review and meta-analysis. Int J Environ Res Public Health. 2020 Jan 1;17:299. doi: 10.3390/ijerph17010299 31906306 PMC6982349

[13] Page MJ, McKenzie JE, Bossuyt PM, Boutron I, Hoffmann TC, Mulrow CD, et al. The PRISMA 2020 statement: an updated guideline for reporting systematic reviews. BMJ. 2021 Mar 29;372:n71. doi: 10.1136/bmj.n71 PMC8005924 33782057

[14] Visualizing Scientific Landscapes [Internet]; [cited 2023 Jul 27]. Available from: https://www.vosviewer.com/

[15] The Ottawa Hospital Research Institute [Internet]. [cited 2023 Jul 27]. Available from: https://www.ohri.ca//programs/clinical_epidemiology/oxford.asp

[16] Critical Appraisal Tools | JBI [Internet]. [cited 2022 Sep 1]. Available from: https://jbi.global/critical-appraisal-tools

[17] Khan MN, Haq ZU, Khan M, Wali S, Baddia F, Rasul S, et al. Prevalence and determinants of violence against health care in the metropolitan city of Peshawar: a cross sectional study. BMC Public Health. 2021 Feb 10;21:330. doi: 10.1186/s12889-021-10243-8 33568108 PMC7877048

[18] Shaikh S, Baig LA, Hashmi I, Khan M, Jamali S, Khan MN, et al. The magnitude and determinants of violence against healthcare workers in Pakistan. BMJ Glob Health. 2020 Apr 15;5:e002112. doi: 10.1136/bmjgh-2019-002112 PMC7199710 32377403

[19] Jafree SR. Workplace violence against women nurses working in two public sector hospitals of Lahore, Pakistan. Nurs Outlook. 2017 Jul-Aug;65:420–427. doi: 10.1016/j.outlook.2017.01.008 28343713

[20] Zafar W, Siddiqui E, Ejaz K, Shehzad MU, Khan UR, Jamali S, et al. Health care personnel and workplace violence in the emergency departments of a volatile metropolis: results from Karachi, Pakistan. J Emerg Med. 2013 Nov; 45:761–772. doi: 10.1016/j.jemermed.2013.04.049. Epub 2013 Sep 4. 24011477 PMC4332856

[21] Qadeer A, Zahid A, Saleem F. Doctors and paramedic staff in Pakistan facing violence and aggression in hospitals. Indo Am J Pharm Sci. 2018 Nov 1;5:11307–11312.

[22] PakMediNet - View Abstract [Internet]. [cited 2022 Sep 1]. Available from: https://www.pakmedinet.com/25371

[23] PakMediNet - View Abstract [Internet]. [cited 2022 Sep 1]. Available from: https://www.pakmedinet.com/35149

[24] PakMediNet - View Abstract [Internet]. [cited 2022 Sep 1]. Available from: https://www.pakmedinet.com/29242

[25] PakMediNet - View Abstract [Internet]. [cited 2022 Sep 1]. Available from: https://www.pakmedinet.com/42147

[26] Khan A, Said AB, Shah BA, Islam F. Violence against nurses at public sector hospital of Peshawar, Pakistan. Int J Innovative Res Dev. 2015;4:4–7.

[27] Ahmed F, Khizar Memon M, Memon S. Violence against doctors, a serious concern for healthcare organizations to ponder about. Ann Med Surg (Lond). 2017 Nov 15;25:3–5. doi: 10.1016/j.amsu.2017.11.003 29255603 PMC5725205

[28] Maheshwari JD, Zaidi SA, Khan K, Iqbal N, Turab SM, Nangrejo R. Association of healthcare workers characteristics with exposure to any form of violence at the hospitals of Karachi. Pak J Med Health Sci. 2022 May 13;16:286–. doi: 10.53350/pjmhs22164286

[29] Somani R, Karmaliani R, Mc Farlane J, Asad N, Hirani S. Sexual harassment towards nurses in Pakistan: are we safe. Int J Nurs Educ. 2015 Apr;7:286–289. doi: 10.5958/0974-9357.2015.00120.8

[30] Siddiqui E, Ejaz K, Razzak JA, Shehzad MU. Prevalence and determinants of violence against emergency medical care providers in Karachi, Pakistan. J Emergency Med. 2013;45:761–772. doi: 10.1016/j.jemermed.2013.04.049 PMC4332856 24011477

[31] Zubairi AJ, Ali M, Sheikh S, Ahmad T. Workplace violence against doctors involved in clinical care at a tertiary care hospital in Pakistan. J Pak Med Assoc. 2019 Sep;69:1355–1359. 31511724

[32] Nayyer-Ul-Islam, Yousuful-Islam M, Farooq MS, Mazharuddin SM, Hussain SA, Umair-Ul-Islam. Workplace violence experienced by doctors working in government hospitals of Karachi. J Coll Physicians Surg Pak. 2014 Sep;24:698–699. 25233981

[33] Zafar W, Khan UR, Siddiqui SA, Jamali S, Razzak JA. Workplace violence and self-reported psychological Health: coping with post-traumatic stress, mental distress, and burnout among physicians working in the emergency departments compared to other specialties in Pakistan. J Emerg Med. 2016 Jan;50:167–77.e1. doi: 10.1016/j.jemermed.2015.02.049 26412103

[34] View of Workplace Violence against Nurses Working in Emergency Departments at Public Hospitals Lahore [Internet]; [cited 2022 Sep 1]. Available from: https://ojs.njhsciences.com/index.php/njhs/article/view/82/76https://ojs.njhsciences.com/index.php/njhs/article/view/82/76

[35] Mirza NM, Amjad AI, Bhatti AB, Tuz Zahra Mirza F, Shaikh KS, Kiani J, et al. Violence and abuse faced by junior physicians in the emergency department from patients and their caretakers: a nationwide study from Pakistan. J Emerg Med. 2012 Jun;42:727–733. doi: 10.1016/j.jemermed.2011.01.029 21669508

[36] Shaikh S, Shahzad H, Khan M, Baig L, Jamali S, Hashmi I, et al. Effect of low-cost interventions to reduce the incidence of violent events in two public sector tertiary-care emergency departments, Pakistan. East Mediterr Health J. 2022 Feb 27;28:144–151. doi: 10.26719/emhj.22.026 35304911

[37] Baig L, Tanzil S, Shaikh S, Hashmi I, Khan MA, Polkowski M. Effectiveness of training on de-escalation of violence and management of aggressive behavior faced by health care providers in a public sector hospital of Karachi. Pak J Med Sci. 2018 Mar-Apr;34:294–299. doi: 10.12669/pjms.342.14432 29805396 PMC5954367

[38] Baig LA, Shaikh S, Polkowski M, Ali SK, Jamali S, Mazharullah L, et al. Violence against health care providers: a mixed-methods study from Karachi, Pakistan. J Emergency Med. 2018 Apr 1;54:558–566. doi: 10.1016/j.jemermed.2017.12.047 29449119

[39] Khan MN, Khan I, Ul-Haq Z, Khan M, Baddia F, Ahmad F, et al. Managing violence against healthcare personnel in the emergency settings of Pakistan: a mixed methods study. BMJ Open. 2021 Jun 1;11:e044213. doi: 10.1136/bmjopen-2020-044213 PMC8208019 34130958

[40] Shahzad A, Malik RK. Workplace violence: an extensive issue for nurses in Pakistan-: a qualitative investigation. J Interpers Violence. 2014 Jul;29:2021–2034. doi: 10.1177/0886260513516005 24390355

[41] Bhatti OA, Rauf H, Aziz N, Martins RS, Khan JA. Violence against healthcare workers during the COVID-19 pandemic: a review of incidents from a lower-middle-income country. Ann Glob Health. 2021 Apr 23;87:41. doi: 10.5334/aogh.3203 33977084 PMC8064297

[42] Njaka S, Edeogu OC, Oko CC, Goni MD, Nkadi N. Work place violence (WPV) against healthcare workers in Africa: a systematic review. Heliyon. 2020 Sep 14;6:e04800. doi: 10.1016/j.heliyon.2020.e04800 32964153 PMC7490814

[43] Hahn S, Zeller A, Needham I, Kok G, Dassen T, Halfens RJ. Patient and visitor violence in general hospitals: a systematic review of the literature. Aggression And Violent Behavior. 2008 Nov 1;13:431–441. doi: 10.1016/j.avb.2008.07.001

[44] Fisekovic Kremic MB, Terzic-Supic ZJ, Santric-Milicevic MM, Trajkovic GZ. Encouraging employees to report verbal violence in primary health care in Serbia: a cross-sectional study. Zdr Varst. 2016 Jul 28;56:11–17. doi: 10.1515/sjph-2017-0002 28289458 PMC5329780

[45] Ramacciati N, Guazzini A, Caldelli R, Rasero L. User-friendly system (a smartphone app) for reporting violent incidents in the emergency department: an Italian multicenter study. Med Lav. 2021 Feb 23;112:68–81. doi: 10.23749/mdl.v112i1.9984. PMID: 33635296; PMCID: PMC8023056. 33635296 PMC8023056

[46] Bhatti MW. JPMC vandalised by attendants of patient suspected of dying from coronavirus [Internet]. Geo News; 2020 [cited 2023 Jul 27]. Available from: https://www.geo.tv/latest/288066-jpmc-vandalised-by-attendants-of-patient-suspected-of-dying-from-coronavirus

[47] Martins RS, Bhatti OA, Mian AI. Violence against Health care workers in Pakistan during the COVID-19 pandemic. JAMA Health Forum. 2020 Oct 1;1:e201263. doi: 10.1001/jamahealthforum.2020.1263 36218555

[48] Shah B. At Lahore’s Mayo Hospital, doctors treating COVID-19 patients face hostility and mistrust [Internet]. Geo News; 2020 [cited 2023 Jul 27]. Available from: https://www.geo.tv/latest/290251-at-lahores-mayo-hospital-doctors-treating-covid-19-patients-face-hostility-and-mistrust

[49] Regan H, Yeung J, Renton A, Woodyatt A, Wagner M. Doctors arrested in Pakistan after protests over lack of PPE. CNN; April 6, 2020 [cited 2023 Jul 27]. Available from: https://edition.cnn.com/world/live-news/coronavirus-pandemic-04-06-20/h_5b2fea9ff8892b69cfe1b006f19c5381

[50] Simons S. Workplace bullying experienced by Massachusetts registered nurses and the relationship to intention to leave the organization. ANS Adv Nurs Sci. 2008 Apr-Jun;31:E48–59. doi: 10.1097/01.ANS.0000319571.37373.d7 18497581

[51] Choiniere JA, MacDonnell JA, Campbell AL, Smele S. Conceptualizing structural violence in the context of mental health nursing. Nurs Inq. 2014 Mar;21:39–50. doi: 10.1111/nin.12028 23517526

[52] Kaya S, Demir İ B, Karsavuran S, Ürek D, İ̇lgün G. Violence against doctors and nurses in hospitals in Turkey. J Forensic Nurs. 2016 Jan-Mar;12:26–34. doi: 10.1097/JFN.0000000000000100 26910266

[53] Jafree SR. Women, healthcare, and violence in Pakistan. Oxford, United Kingdom: Oxford University Press; 2018.

[54] Bano S, Pradhan NA, Rizvi N, Midhet F. Nurses’job dissatisfaction and associated factors at public sector tertiary care hospital in Pakistan: a qualitative study. J Nurs. 2019 May 1;9:9. doi: 10.26634/jnur.9.2.16352

[55] Class divide — Pakistan’s main problem [Internet]; 2018 [cited 2023 Jul 27]. Available from: https://dailytimes.com.pk/12847/class-divide-pakistans-main-problem/

[56] Kahsay WG, Negarandeh R, Dehghan Nayeri N, Hasanpour M. Sexual harassment against female nurses: a systematic review. BMC Nurs. 2020 Jun;29;19:58. doi: 10.1186/s12912-020-00450-w. Erratum in: BMC Nurs. 2020 Jul 13;19:64. PMC7324991 32612455

[57] Karmaliani R, Pasha A, Hirani S, Somani R, Hirani S, Asad N, et al. Violence against women in Pakistan: contributing factors and new interventions. Issues Ment Health Nurs. 2012 Dec;33:820–826. 23215983 10.3109/01612840.2012.718046

[58] Arnetz JE. The joint commission’s New and revised workplace violence prevention standards for hospitals: a major step forward toward improved quality and safety. Jt Comm J Qual Patient Saf. 2022 Apr;48:241–245. doi: 10.1016/j.jcjq.2022.02.001. Epub 2022 Feb 5. PMID: 35193809; PMCID: PMC8816837. 35193809 PMC8816837

[59] Herrmann A, Seubert C, Glaser J. Consequences of exposure to violence, aggression, and sexual harassment in private security work: a mediation model. J Interpers Violence. 2022 Jun;37:NP9684–NP9711. doi: 10.1177/0886260520984432 . Epub 2020 Dec 31. PMID: 33380234; PMCID: PMC9136388. 33380234 PMC9136388

[60] Arbury S, Hodgson M, Zankowski D, Lipscomb J. Workplace violence training programs for Health care workers: an analysis of program elements. Workplace Health Saf. 2017 Jun;65:266–272. doi: 10.1177/2165079916671534 . Epub 2017 Mar 9. Erratum in: Workplace Health Saf. 2017 Aug;65(8):380. 28557640

[61] Guidelines for preventing workplace violence for health care & social service workers. Washington (DC): U.S. Dept. of Labor, Occupational Safety and Health Administration; 2004.

