# Supplementary material for: Workplace violence against healthcare workers in Pakistan; call for action, if not now, then when? A systematic review
Source: Glob Health Action. 2023 Nov 8;16(1):2273623. doi: 10.1080/16549716.2023.2273623 (PMC10653705; doi:10.1080/16549716.2023.2273623)
Supplement: Supplemental Material [file ZGHA_A_2273623_SM0667.docx]

**Supplementary Appendix**

**Table 1 - Detailed Search Strategy**

| **Database** | **String** | **Results** |
| --- | --- | --- |
| Pubmed | ((("health personnel"[MeSH Terms] OR ("health"[All Fields] AND "personnel"[All Fields]) OR "health personnel"[All Fields] OR ("healthcare"[All Fields] AND "workers"[All Fields]) OR "healthcare workers"[All Fields] OR ("nurse s"[All Fields] OR "nurses"[MeSH Terms] OR "nurses"[All Fields] OR "nurse"[All Fields] OR "nurses s"[All Fields]) OR ("doctor s"[All Fields] OR "doctoral"[All Fields] OR "doctorally"[All Fields] OR "doctorate"[All Fields] OR "doctorates"[All Fields] OR "doctoring"[All Fields] OR "physicians"[MeSH Terms] OR "physicians"[All Fields] OR "doctor"[All Fields] OR "doctors"[All Fields]) OR (("support"[All Fields] OR "support s"[All Fields] OR "supported"[All Fields] OR "supporter"[All Fields] OR "supporter s"[All Fields] OR "supporters"[All Fields] OR "supporting"[All Fields] OR "supportive"[All Fields] OR "supportiveness"[All Fields] OR "supports"[All Fields]) AND ("staff"[All Fields] OR "staff s"[All Fields] OR "staffs"[All Fields]))) AND ("violence"[MeSH Terms] OR "violence"[All Fields] OR "violence s"[All Fields] OR "violences"[All Fields])) OR ("abusable"[All Fields] OR "abuse s"[All Fields] OR "abused"[All Fields] OR "abuser"[All Fields] OR "abuser s"[All Fields] OR "abusers"[All Fields] OR "abuses"[All Fields] OR "abusing"[All Fields] OR "abusive"[All Fields] OR "abusively"[All Fields] OR "abusiveness"[All Fields] OR "substance related disorders"[MeSH Terms] OR ("substance related"[All Fields] AND "disorders"[All Fields]) OR "substance related disorders"[All Fields] OR "abuse"[All Fields]) OR ("harass"[All Fields] OR "harassed"[All Fields] OR "harasser"[All Fields] OR "harassers"[All Fields] OR "harassing"[All Fields] OR "harassment"[All Fields] OR "harassments"[All Fields]) OR ("assault"[All Fields] OR "assaulted"[All Fields] OR "assaulter"[All Fields] OR "assaulters"[All Fields] OR "assaulting"[All Fields] OR "assaultive"[All Fields] OR "assaultiveness"[All Fields] OR "assaults"[All Fields])) AND ("pakistan"[MeSH Terms] OR "pakistan"[All Fields] OR "pakistan s"[All Fields]) | 926 |
| Cochrane CENTRAL | (((violence) OR (threat*) OR (abuse*)) AND ((healthcare workers*) OR (nurses) AND (pakistan hospitals) OR (pakistani hospitals))) | 81 |
| Google  Scholar | "healthcare workers" AND "violence" OR "aggression" AND "Pakistan" OR "Pakistani hospitals" | 5320 |
| Embase | (((((healthcare workers or healthcare personnel) and violence) or abuse) and pakistan) or pakistan hospitals) | 631 |
| Science Direct | ((("healthcare staff" OR "healthcare workers") AND ("violence" OR "harassment" OR "abuse") AND (pakistan hospitals OR pakistani hospitals))) | 216 |
| ERIC | healthcare violence workplace violence pakistan pakistani hospitals | 168 |

Table 2. Study characteristics

| **Author (Year)** | | **Type of Study** | **Region** | **Type of violence/s assessed** | **Mode/s of violence assessment** | **Perpetrator** | **Outcomes** |
| --- | --- | --- | --- | --- | --- | --- | --- |
|  | Shaikh 2022^18^ | Pre-Post Quasi-Experimental | Karachi, Peshawar | Physical, Verbal | - | Pre-Intervention; No of events-  HCW: 14  Patient/ attendant: 72  Mob: 4  Pre-Intervention-  HCW: 14  Patient/ attendant: 29  Mob: 2 | - The prevalence of verbal violence was higher compared to physical violence. |
|  | Khan 2021^19^ | Cross-sectional | Peshawar | Verbal, Physical, and Combined | ILO, WHO designed scale for workplace violence assessment | Attendant/relative of the patient: 44%, patients: 20%  In 85% of incidents, two or more perpetrators were involved in each event. | - Emergency departments (34%) and wards (30%) were the common sites of violent incidents in the healthcare facilities - A higher prevalence of violence was observed in public healthcare facilities, and among physicians and support staff compared to nurses and paramedics |
|  | Shaikh 2020^20^ | Cross-sectional survey | Islamabad, Peshawar, Lahore, Karachi | Physical, Verbal, facility damage, harassment, False accusations, Robbery, and extortion | Self-designed questionnaire | Attendants: 67.7-90.5%  Patients: 6.7-14.4% | - Among hospital HCWs, those working in private hospitals were significantly less likely to experience physical violence (p=0.001) and verbal violence (p=0.001) - Female HCWs were significantly more likely to experience verbal violence. - Among the different types of fields HCWs, emergency vehicle operators were significantly more likely to experience verbal violence with reference to LHWs (adjusted OR=1.97; 95% CI 1.31 to 2.94; p=0.001). - HCWs in emergency departments were significantly more likely to experience physical violence (adjusted OR=5.84; 95% CI 2.17 to 15.72; p<0.001). - Among the different types of HCWs, in comparison with administration staff, security guards were significantly more likely to experience verbal violence (adjusted OR=1.77; 95% CI 1.07 to 2.92; p=0.024). |
|  | Jafree 2017^21^ | Cross-sectional survey | Lahore | Physical, verbal, sexual | WHO designed questionnaire | PV-  Patient: 34.7%  Attendant: 23%  Coworker: 53.4%  Combination: 2.8%  VV-  Patient: 33.6%  Attendant: 38.5%  Coworker: 2.5%  Combination: 25.4%  SV-  Patient: 25.5%  Attendant: 5.1%  Coworker: 32.8%  Combination: 25.5% | - 73.1% of the nurses experienced some sort of violence in the past year. - SV was more likely to occur among single nurses (AOR: 1.98, 95 % CI: 0.41–2.35), those belonging to non-Punjabi ethnicity (AOR: 1.69, 95 % CI: 12 0.11–3.30), and Muslims (AOR: 1.95, 95 % CI: 0.43–2.12). - Staff and student nurses had higher odds of experiencing PV (AOR: 1.06, 95 % CI: 0.25–2.07), VV (AOR: 1.53, 95 % CI: 0.76–2.71) & SV (AOR: 1.75, 95 % CI: 0.59–2.55). - Nurses working the night or evening shift had 17 times higher odds of experiencing VV & SV (AOR: 2.08, 95 % CI: 1.70–3.23 & AOR: 2.14, 95 % CI: 0.96–3.88, respectively). |
|  | Zafar 2013^22^ | Cross-sectional survey | Karachi | Physical, verbal | WHO designed questionnaire | PV-  Patients’ relatives: 64%,  Patient: 20.4%  VV: Patients’ relative: 70%,  Patient: 16.1% | - Among those who reported being physically attacked, 54.5% were males, 50% were younger than 30 years, 59% were nurses, 41% were physicians, 50% had experience of 5 years or less in the health sector, and half were employed in public and half in the private sector hospitals. |
|  | Qadeer 2018^23^ | Cross-sectional | Lahore | Physical aggression, threats, verbal attacks, harassment, verbal and physical abuse | N/A | Patient's family: 68%  Patients:  31% | - Violent abuse was the most frequently experienced type of abuse. |
|  | Lubna 2014^24^ | Cross-sectional | Islamabad | Physical, verbal, bullying,  and sexual | N/A | Patient/Client/Manager/ Supervisor: 27.6%  Patient relatives: 44%  Outsiders: 10.6%  Staff Members: 93.8%  Worker: 6.6% | - Verbal abuse, bullying, and sexual harassment were significantly linked with qualification (p <0.05). - Nurses enrolled in diploma programs were more likely to experience harassment as compared to BScN nurses. |
|  | Maaari 2017^25^ | Cross-sectional | Karachi | Physical attacks, verbal abuse, bullying, sexual harassment | ILO/ICN/WHO/PSI joint program tool | Patient: 33.3%  Relative of the patient: 52.3%  Staff Member:  9.5%  Manager/Supervisor: 2.3% | - 46.7% of the nurses reported that they had been physically assaulted in the previous year. Of those who reported being physically assaulted, 57% were females, 51% were between the ages of 25 and 39, 59% were nurses, and 24% were in management jobs. In addition, 40% of the victims had work experience of 1 to 5 years in the health sector. - 52.2% of the nurses reported verbal abuse during the previous 12 months. 55% of those verbally abused were female, 68% were staff nurses, and 60% had experience working in the healthcare industry for 6 to 10 years. - 27.8% said they had experienced bullying the previous year. 60 percent of them were female, 40 percent were in their 25th to 34th year of life, 68 percent were staff nurses, and 57 percent had one to five years of work experience. - 15.6% of nurses said they had experienced sexual harassment in the previous 12 months. Of those, 78.5% were female, 70% were under 25 years old, 78% worked as nurses, and 57% had between one and five years of experience in the healthcare industry. |
|  | Imran 2013^26^ | Cross-sectional | Lahore | Verbal abuse, physical abuse | Self-administered questionnaire | Patients’ relatives: 71%  Patient: 30.5%  Coworkers: 52.8%  Others: 3.3% | - In the emergency room, there were violent accidents in 76.8% of cases. The ward accounted for 30.5% of these acts, and the outdoors for 10.7% of the cases. |
|  | Din 2019^27^ | Cross-sectional | Faisalabad | Verbal abuse, the threat to assault, physical assault | A pre-designed and pre-tested questionnaire | N/A | - WPV is found to be more prevalent in younger doctors (85.9%) in the 24-34 years age group. |
|  | Khan 2015^28^ | Cross-sectional | Peshawar | Physical and verbal violence | Self-designed questionnaire | Patients: 35%  Patient relatives: 30%  Staff members: 20% | - Ninety-one percent of these violent occurrences took place within hospitals.  Predominantly, these violent acts of aggression were carried out in medical facilities (91%) |
|  | Ahmed 2018^29^ | Cross-sectional survey | Karachi | Verbal abuse, Threats, Intimidation, Sexual harassment, Physical attacks, Weaponry attacks, Theft, Damage to family or property | Self-designed questionnaire | N/A | - When compared to male doctors, who reported feeling "safe," only 70.39% (n = 107) of female doctors did (p = 0.001, CI = 95%). - 10% (n = 37) of female respondents chose the "somewhat safe" or "partially safe-partially unsafe" option, compared to 23% of male respondents. In comparison to 7.2% of males, 42.20% of females reported having "less sense of safety to not at all safe" (p = 0.001, CI = 95%). - The percentage of females who felt safe during calls dropped substantially from 75% to 7.2%. |
|  | Shahzad 2014^30^ | Qualitative investigation | Islamabad | Verbal and physical abuse | WHO designed questionnaire | N/A | - Verbal violence was the most frequently experienced type of WPV, and younger nurses (30 years and younger) were mostly affected. - Verbal violence was most prevalent in morning shifts, and physical violence during night shifts. |
|  | Maheshwari 2022^31^ | Cross-sectional | Karachi | Not specified | WHO Survey | N/A | - The likelihood of WPV among male healthcare professionals was somewhat higher than that of their female counterparts, although this difference was not statistically significant. - Practitioners with more than five years of experience (83.7%) and full-time employees (75.6%) reported a significant proportion of violence in the workplace. CMO/MO had reported encountering greater workplace violence (92.7%), followed by surgeons (81.0%) and physicians (79.2%) - Violence was more prevalent among HCWs with more than five years of experience than among those with fewer than five years. |
|  | Somani 2015^32^ | Cross-sectional | Karachi | Verbal abuse, bullying, sexual harassment, and racial harassment | Workplace violence in the health sector country case studies research instrument (2003) | Patient’s relatives: 47.8%  Staff Members: 32.6% | - Sexual harassment was most common among nurses between the ages of 19 and 29, with a reported incidence of 54.4% (n=25/46) in this age group. Similar to this, female nurses were more likely than male nurses to experience these types of violence (89.1% n=41/46 female versus 10.9% n=05/46male). - 95.7% of the 46 nurses who experienced sexual assault were either staff nurses or nursing interns. - Sexual harassment was prevalent (at a rate of 50%) among nurses with fewer than five years of work experience. In a related manner, when compared to nurses who worked fixed shifts, nurses who performed shift duties reported a higher occurrence of sexual harassment, ranging from 80% to 85%. |
|  | Siddiqui 2010^33^ | Cross-sectional | Karachi | Physical and verbal abuse | N/A | N/A | - Physician employment (OR 2.06; 95% CI 1.06 to 4.02) and having less than five years of work experience (OR 2.41; 95% CI 1.05 to 5.57) were found to be predictive of violence. |
|  | Zubairi 2019^34^ | Cross-sectional | Karachi | Physical, verbal, sexual abuse, racial, threat, bullying | ILO, ICN, WHO, & PSI | Faculty: 28%  Patients/ Attendants: 27%  Co-trainees: 21% | - The overall prevalence of workplace violence was 53.4%. - Trainees were more exposed to workplace violence compared to faculty members. (p<0.01) - Females experienced a higher degree of sexual harassment compared to their male counterparts. (p=0.04) - Emergency medicine physicians had the highest exposure to verbal abuse. (p<0.01) |
|  | Islam 2014^35^ | Cross-sectional | Karachi | Physical, verbal, sexual | Adapted from a Swedish study^++^ | Attendants: 61.5%  Patients: 14.7%   Mob: 14.7%  Others: 8.3% | - Overall, 74.9% of participants had experienced some sort of workplace violence. - Females experienced more violence than their male counterparts for keeping the patients/attendants waiting for longer times (p = 0.028). - Men experienced more politically targeted violence when compared to women. (p < 0.001) |
|  | Zafar 2015^36^ | Cross-sectional | Karachi | Physical, verbal | ILO/ICN/WHO/PSI joint Program | VV  Patients: 22  Attendants: 77  Coworkers: 6  PV- Patients: 4  Attendants: 19  Coworkers: 2  Others: 3 | - Pathology was the least likely specialty to report any form of violence when compared to the rest. - Other specialties like emergency medicine, internal medicine, surgery, and pediatrics had comparable rates of experiencing workplace violence. (p=0.02) |
|  | Ijaz 2018^37^ | Cross-sectional | Lahore | Physical, verbal, sexual | Adapted from a Saudi study + | PV- Patients: 12  Attendants: 20  Coworkers: 114  Nonphysical-  Patients: 29  Attendants: 93  Coworkers: 8 | - Physical violence was encountered less frequently compared to nonphysical violence. 13.6% of the nurses experienced it once, while 7.48% experienced it 2-3 times in the past year. 1.3% had even been exposed 4-5 times in the previous year. |
|  | Mirza 2011^38^ | Cross-sectional | Nationwide | Physical, verbal | Self-designed questionnaire | PV-  Patients: 39  Attendants: 119  VV-  Patients: 498  Attendants: 1004 | - 519 (76.9%) participants reported some form of physical or verbal abuse. - Male physicians were more likely to face verbal and physical abuse from patients/ attendants than female physicians (p < 0.05) |
|  | Baig 2018^39^ | Mixed methods (cross-sectional + qualitative) | Karachi | Physical, verbal | N/A | Attendants: 58.1% Public: 26% | - Overall, 275 (33.5%) participants experienced violence. - Physicians, security staff, and ambulance staff reported the highest frequency of experiencing verbal violence (p = 0.004). - Women were less likely to experience physical violence than men (p = 0.001). |
|  | Baig 2018 (2) ^40^ | Quasi-experiment | Karachi | - | - | Intervention:  Patient-1  Attendant-42  Control:  Patient-1  Attendant-42 | - 17 (23.9%) people in the intervention group and 17 (24. 3%) participants in the control group experienced some sort of violence |
|  | Khan 2021 (2) ^41^ | Mixed methods study | Peshawar | Physical, verbal | ILO/ICN/WHO/PSI Joint Program | Intervention:  Attendants: 87%  General public: 1%  Hospital staff: 2%  Combination: 3%  Control group:  Patient: 5%  Attendants: 76%  General public: 4%  Combination: 5% | - Verbal violence was reported more commonly than physical violence. |

ILO: International Labor Office

WHO: World Health Organization

WPV: Workplace Violence

ICN: International Council of Nurses

PSI: Public Services International

Reported for a limited no. of cases only*

- Alshehri, Fuaad Ali. “Workplace violence against nurses working in emergency departments in Saudi Arabia: a cross-sectional study.” (2017).

++ Judith EA, Bengt BA. Implementation and evaluation of a practical intervention program for dealing with violence towards health care workers. J Adv Nursing 2000; 31:668-80.

Table 3: Population characteristics

| **Author (Year)** | **Total Population (N),**  **Job Category (n)** | **Type of Health facility (n)** | **Sex,**  **Age (year); Mean+SD** | **Prevalence of Verbal violence (%)** | **Prevalence of Physical violence (%)** | **Any other violence (%)** |
| --- | --- | --- | --- | --- | --- | --- |
| Shaikh 2022^18^ | Pre/ Post-Intervention-256/ 225^*^;  Doctor: 64/ 85  Nurse: 64/ 42  Guard: 92/ 62  Admin: 11/ 16 | Public tertiary care emergency depts: 2 | - | No. of events-  Pre-intervention: 336  Post-intervention: 268 | No. of events-  Pre-intervention: 54  Post-intervention: 28 | Overall events-  Pre-intervention: 346  Post-intervention: 270 |
| Khan 2021^19^ | N= 842  Physicians: 172  Nurses: 193  Paramedics: 215  Support staff: 262 | Public: 694  Private:148 | Male:574,  Female:268,  Age: Exposed group: 35.44+9.2  Non-exposed: 35.52+10.0 | 45 | 0.7 | Combination of physical, verbal, and facility damage: 22  Combination of physical and verbal abuse: 23  Facility damage: 0.6 |
| Shaikh 2020^20^ | 8579,  Doctors, nurses, technicians, support staff, ambulance staff, vaccinators, lady health workers, midwives, lady health visitors | Tertiary care hospitals, Primary care hospitals, Private clinics | Male: 55.6%  Female: 44.4%  Age: 33.19+8.86 | Experienced: 33.9 | Experienced: 6.6 | Facility Damage:  0.4,  Bullied/Harassed:1.1,  Falsely accused: 2.2,  Robbed: 0.3,  Extorted: 0.1 |
| Jafree 2017^21^ | 309  Nurses | Tertiary care public hospitals | Female: 100%  Age: 34.8+10.13 | 57.3 | 53.4 | Sexual violence: 26.9 |
| Zafar 2013^22^ | 266,  Physicians:134, Nurses: 132 working in ED | Private | Males:146  Females: 120;  Age (n%): <30: 62.8  30–39: 27.1  40–49: 7.9  50–59:2.2 | 72.5 | 16.5 | N/A |
| Qadeer 2018^23^ | 150  Doctors: 120  Nurses: 25  Medical & Allied, Surgery & Allied, Emergency Department | Public hospitals | Age: 30 ± 5.02; Male (n = 93, 62%)  Female (n=57  66.66%) | N/A | 67 | N/A |
| Lubna 2014^24^ | 300  Nurses  General nursing, midwifery, BSN, nursing students | Public and Private hospitals | Age: 20-29 | 63 | 17 | Bullying: 16  Sexual harassment: 4 |
| Maari 2017^25^ | 90  ICU and ED Nurses | Tertiary Care hospital | Age: < 49  Female: 54 (60%) | 52.2 | 46.7 | Bullying: 27.8  Sexual harassment: 15.6 |
| Imran 2013^26^ | 164  Doctors: 135  Nurses: 29 | Public hospital | Age: 30.58±8.02  Males (n=102, 62.2%) | 86 | N/A | Threats: 34.7 |
| Din 2019^27^ | 185  Consultant: 32  Postgraduate resident: 72  House Officer: 81 | Public teaching hospital | Age: > 24  Males: 127 (68.6%)  Females: 58 (31.4%) | 76 | 6 | Threat to assault: 18 |
| Khan 2015^28^ | 70  Staff nurses: 51  Student nurses: 14  Head nurses: 48 | Public Hospital | Age: < 50  Female:  67 (95.7%)  Male: 03 (4.3%) | 67.4 | 29 | N/A |
| Ahmed 2018^29^ | 769  Physicians | Public and private hospitals | Age: 31 ± 7.68  Females: 545 (71%)  Males: 224 (29%) | N/A | N/A | Mild aggression: 85.11%  Moderate incidents: 62%  Severe violence: 38.1% |
| Shahzad 2014^30^ | 20  Nurses | Public hospitals | Age: > 40  Male: 05 (25%)  Female: 15 (75%) | 100 | 20 | N/A |
| Maheshwari 2022^31^ | 300  Physician, Surgeon, Chief Medical officer, Medical officer, PG resident, house officer | Public hospitals | Age: > 35  Female: 112 (70.9%)  Male: 111 (78.2%) | N/A | N/A | N/A |
| Somani 2015^32^ | 458  In-patient department and ED nurses | Public and private hospitals | N/A | N/A | N/A | Sexual Harassment: 10 |
| Siddiqui 2010^33^ | 384  Physicians, registered nurses, nursing aids, porters, guards, unit receptionists, and housekeepers | Tertiary care hospitals | Age: N/A  Males: 250 (65%)  Females: 134 (34.8%) | 25 | 5.9 | N/A |
| Zubairi 2019^34^ | 185  Trainee doctors- 121  Faculty members- 64 | Tertiary care public hospital | Age: 32.6 ± 8.5  Males: 103 (55.7%)  Females: 82 (44.3%) | 41.6 | 2.7 | Bullying: 28.1  Threat: 24.9  Racial harassment: 7.6  Sexual harassment: 2.7 |
| Islam 2014^35^ | 354  doctors | Tertiary care Public- 3 | Males: 176 (49.7%)  Females:177 (50%)  Age: N/A | 93.2 | 15.1 | Sexual harassment: 3.0  Property damage: 46.0 |
| Zafar 2015^36^ | 179  doctors | Tertiary care Public- 3  Private- 1 | Age: <30: 117 (65.36%)  >30: 62 (34.63%)  Males: 74 (41.3%)  Females: 105 (58.6%) | 60.9 | Experienced-15.6 | - |
| Ijaz 2018^37^ | 147  nurses | Tertiary care public- 2 | Age: <25: 15 (10.2%)  26-40: 128 (87.1%)  >40: 4 (2.7%)  Males: 10 (6.80%)  Females:  137 (93.20%) | 36.1 | 22.44 | Sexual harassment:2  Threat: 50.3 |
| Mirza 2011^38^ | 675  doctors | Public sector tertiary care- 9 | Male 360 (53.3) Female 315 (46.7) | 84.6 | 15.4 | - |
| Baig 2018^39^ | 822  Nurses: 174  Physicians: 124  Technicians:128  Security: 78  Ambulance staff: 115  Support staff: 128 | Public and private, NGOs offering health services | Mean age:  34.51 years  Males: 59.7%  Females: 40.3% | Experienced-30.5,  Experienced or witnessed- 58.5 | Experienced- 14.6,  Experienced or witnessed- 28.6 | Facility damage: 6.2 |
| Baig 2018 (2)^40^ | 141  Intervention; 71  Doctor: 50  Nurse: 11  Medical student: 10  Control- 70  Doctor: 54  Nurse: 14  Medical student: 2 | Tertiary care hospital | Intervention: Age: 27.34+ 6.17 y  Males: 25 (35.2%)  Females: 46 (64.8%):  Control:  29.86 (8.55%)  Males: 24 (34.3%)  Females:  46 (65.7%) | - | - | - |
| Khan 2021 (2)^41^ | 200  Doctors: 10  Nurses: 60  Paramedics: 55  Support staff: 62  Administration: 13 | Tertiary care- 2 | Intervention:  33.52 (9.5)  Males: 73 (73%)  Females: 27 (27%)  Control:  31.62 (8.4) y  Males; 70 (70%)  Females: 30 (30%) | Intervention: 54, Control: 47 | Intervention: 5  Control: 9 | Verbal + Physical  Intervention: 34  Control: 34 |

^*^Total no of participants also includes the patients and their companions.


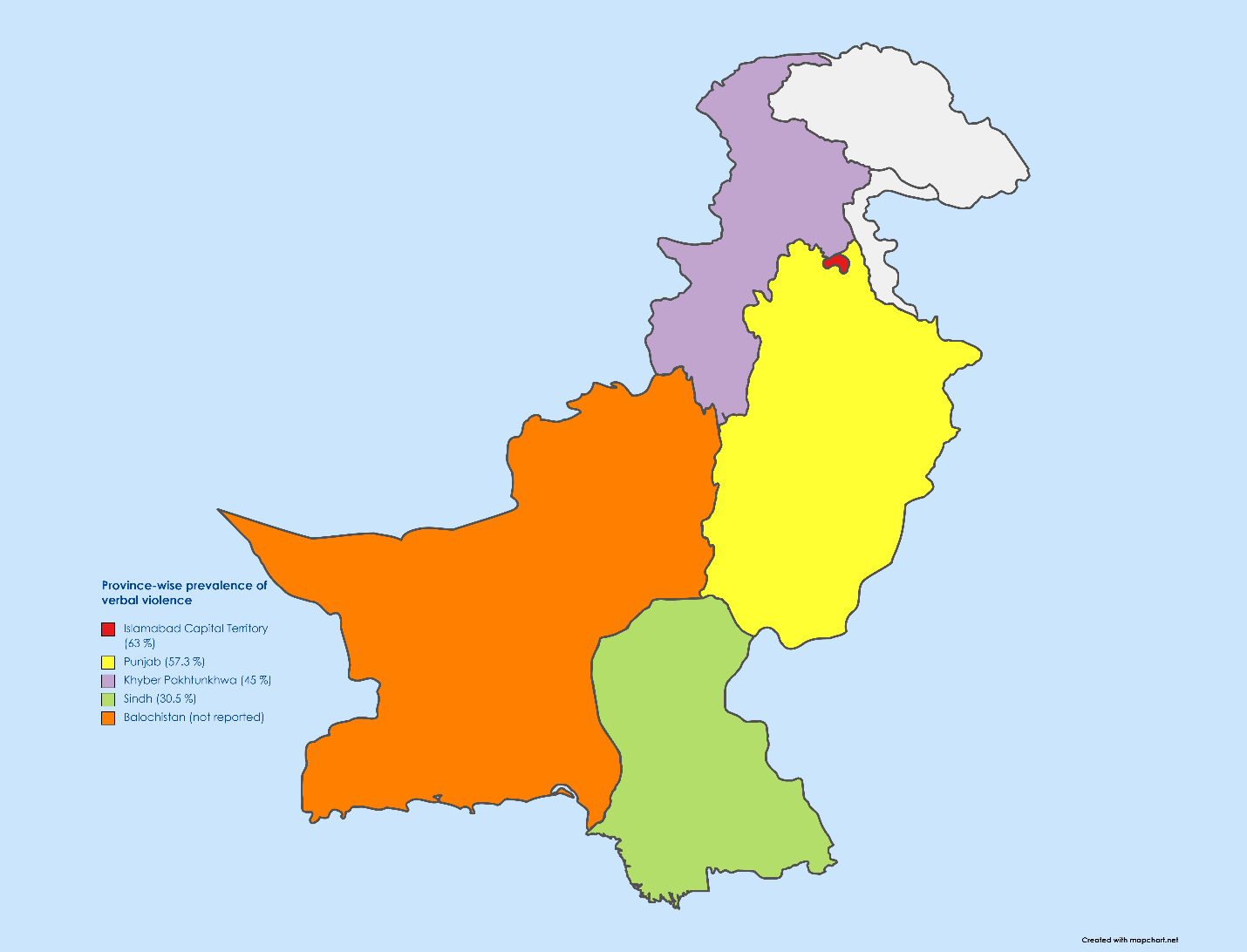


**Figure S1. Provincial prevalence of verbal violence against HCWs in Pakistan^19,21,24,39^**
